# Preterm Life in Sterile Conditions: A Study on Preterm, Germ-Free Piglets

**DOI:** 10.3389/fimmu.2018.00220

**Published:** 2018-02-14

**Authors:** Alla Splichalova, Vera Slavikova, Zdislava Splichalova, Igor Splichal

**Affiliations:** ^1^Laboratory of Gnotobiology, Institute of Microbiology of the Czech Academy of Sciences, Novy Hradek, Czechia

**Keywords:** preterm, enterocyte, intestine, germ-free, piglet, tight junctions, receptors, cytokines

## Abstract

Preterm infants born with immature organ systems, which can impede normal development, can also be highly sensitive to different biological and/or environmental factors. Animal models could aid in investigating and understanding the effects of different conditions on the health of these immunocompromised infants. The epitheliochorial placentation of the pig prevents the prenatal transfer of protective colostral immunoglobulins. Surgical colostrum-deprived piglets are free of maternal immunoglobulins, and the cells that are normally provided *via* colostrum. We bred preterm germ-free piglets in sterile conditions and compared them with their term counterparts. Enterocyte development and intestinal morphology, tight junction proteins claudin-1 and occludin, pattern-recognizing receptors, adaptor molecules and coreceptors (RAGE, TLR2, TLR4, TLR9, MyD88, TRIF, MD2, and CD14), and inflammasome NLRP3 transcription were all evaluated. The production of inflammatory mediators IFN-α, IL-4, IL-6, IL-8, IL-10, IL-12/23 p40, TNF-α, IFN-γ, and high mobility group box 1 (HMGB1) in the intestine of germ-free piglets was also assessed. In the preterm germ-free piglets, the ileum showed decreased lamina propria cellularity, reduced villous height, and thinner and less distinct stratification – especially muscle layer, in comparison with their term counterparts. Claudin-1 transcription increased in the intestine of the preterm piglets. The transcription levels of pattern-recognizing receptors and adaptor molecules showed ambiguous trends between the groups. The levels of IL-6, IL-8, IL-10, and TNF-α were increased in the preterm ileum numerically (though not significantly), with statistically significant increases in the colon. Additionally, IL-12/23 p40 and IFN-γ were statistically significantly higher in the preterm colon. Both blood plasma and intestinal HMGB1 levels were nonsignificantly higher in the preterm group. We propose that the intestine of the preterm germ-free piglets showed “mild inflammation in sterile conditions.” This model, which establishes preterm, hysterectomy-derived germ-free piglets, without protective maternal immunoglobulins, can be used to study influences of microbiota, nutrition, and therapeutic interventions on the development and health of vulnerable immunocompromised preterm infants.

## Introduction

A mammalian fetus develops in the amniotic cavity and is nourished *via* umbilical blood through the placenta (1). Swallowing of the amniotic fluid begins halfway through pregnancy, and the fetal gastrointestinal tract (GIT) fills with amniotic fluid (2). The fetal lungs prepare for postnatal life through fetal breathing movements that inhale and exhale amniotic fluid (3). After birth, the conditions dramatically change, and the newborn suddenly finds itself in dry surroundings. It receives nourishment by liquid diet through the GIT, and gas exchange occurs by breathing through the lungs. Additionally, microbiota settle the GIT, stimulate the immune system of the newborn host, and produce metabolites that are often essential for the host—e.g., K and B group vitamins (4).

A full-term human gestation from conception to birth is about 38 weeks. Preterm deliveries comprise 11% of all deliveries, but this number is increasing in most countries (5). The preterm neonates are usually capable of oral feeding, but if they are born at the gestational age of 34 weeks or less, they need breathing support, because maturation of respiration is still in progress (6). While vaginally born infants are colonized by vaginal and fecal microbiota of the mother immediately during birth (7), infants born *via* Cesarean section are colonized by microbiota from the hospital environment (8). Preterm infants with underdeveloped immune systems and unestablished balanced microbiota, however, are at a very high risk of dysbiosis that often preceding development of necrotizing enterocolitis (NEC) (9). Controlled colonization of the GIT newborns may be one of the ways to establish balanced microbiota that is necessary for healthy development (10, 11).

The *in vivo* studies of crosstalk of the gastrointestinal microbiota, nutrition, and underdeveloped immune system of the vulnerable preterm newborns require the use of suitable animal models of the preterm host. The pig shows closer anatomical, physiological, and genetic similarities to the human than the frequently used laboratory rodents (12). The preterm piglet model has been used to study conditions of the development of NEC (13, 14) and the immunity of preterm infants (15). In contrast to the hemochorial placenta of the human, the epitheliochorial placenta of the pig prevents the prenatal transfer of immunoglobulins that passively immunize fetuses (1, 16). The surgery-derived colostrum-deprived germ-free piglets lack postnatal intake of colostral immunoglobulins (17) and immune cells (18). These immunocompromised piglets (19–21) lend themselves to studies of host-microbiota crosstalk and bacterial interferences. Pillar study in germ-free piglets colonized with infant microbiota has been reported recently (22).

Pattern recognition receptors (PRRs) are receptors that recognize molecular structures representative of microbes termed pathogen-associated molecular patterns (PAMPs) (23). The PRRs include C-type lectin receptors, NOD-like receptors (NLRs), retinoid acid-inducible gene I-like receptors, and toll-like receptors (TLRs). PRRs mediate downstream signaling pathways, leading to the activation of nuclear factor kappa B (NF-κB), mitogen-activated protein kinase (MAPK), or cleaving by caspases. They result in the production of inflammatory cytokines or type I interferons (24, 25). TLR2 recognizes a cell wall component of Gram-positive bacteria peptidoglycan, while TLR4 recognizes a cell wall component of Gram-negative bacteria lipopolysaccharide. TLR5 recognizes a flagellin that is a protein of bacterial flagella, and TLR9 recognizes microbes commonly associated with unmethylated CpG dinucleotides. Some of the bacterial structures are bound to compound TLRs—i.e., triacyl lipopeptides to TLR2/TLR1 and lipoteichoic acid to TLR2/TLR6 (26). The cytosolic NLRs NOD1 and NOD2 detect the intracellular presence of the peptidoglycan fragments meso-diaminopimelic acid and muramyl dipeptide, respectively (24). Large intracellular complexes, inflammasomes, contain another set of NLRs that trigger a cascade, leading to the maturation of IL-1β and IL-18 by caspase cleaving (27). Mechanical trauma, burns, ischemia, myocardial infarction, cancer, autoimmune diseases, and other non-infectious states lead to tissue damage and cell necrosis, resulting in the release of damage-associated molecular patterns (DAMPs). In contrast to foreign PAMPs, DAMPs are the host body’s own structures and their release provokes sterile inflammation (28, 29) that can lead to consequences similar to those of sepsis (30). High mobility group box 1 (HMGB1), a component of DAMPs, is a nuclear DNA-binding protein necessary for transcription and released in cell necrosis, or actively from macrophages after their stimulation (31). Certain TLRs and NLRs are involved in both PAMPs and DAMPs recognition and signaling pathways (32). In the intestine, PRRs reside mainly on and/or in enterocytes and immune cells of the lamina propria (33). Pillar tissue-specific TLR transcription in germ-free and conventional piglets has been reported recently (33, 34).

We hypothesize that the GIT of preterm newborns is inflamed and DAMPs, *via* PRR signaling pathways, induce inflammatory mediators, such as cytokines. In this work, we evaluated the possible development of sterile inflammation in a germ-free piglet model of preterm infants. Enterocyte maturity, intestinal barrier TJ proteins (claudin-1 and occludin), transcription of pattern recognizing receptor pathways (RAGE, TLR2, TLR4, TLR9, MyD88, TRIF, MD2, CD14, and inflammasome NLRP3), the production of inflammatory cytokines, and production of inflammatory mediators (IFN–α, IL–4, IL-6, IL-8, IL-10, IL–12/23 p40, TNF-α, IFN-γ, and HMGB1) in the GIT of preterm germ-free piglets were studied to evaluate a potential preterm germ-free piglet model of immunocompromised preterm infants.

## Materials and Methods

### Ethical Statement

Experiments with animals were approved by the Animal Care and Use Committee of the Institute of Microbiology in accordance with local and European laws for the protection of animals. The number of piglets in the preterm group was higher to accommodate for unexpected events, but the number of the term piglets was reduced to a number that was sufficient for statistical comparison between groups.

### Term Germ-Free Piglets

A conventional miniature pig herd in the Animal Research Institute (Kostelec nad Orlici, Czech Republic) was monitored for transplacentally transmitted viral (parvoviruses, Aujezsky’s Disease virus, PRRS), bacterial (leptospires, brucellosis), and protozoan (toxoplasmosis) pathogens. Pregnant sows were orally treated with 20 mg of altrenogest (Regumate^®^ Porcine; MSD Animal Health BVBA, Brussel, Belgium) per day for 18 days to synchronize their conception. Vitamins A (300,000 IU/ml), D (100,000 IU/ml), and E (50 mg/ml) (ADE-oleosum; Veyx-Pharma GmbH, Schwarzenborn, Germany) were intramuscularly (i.m.) administered at 0.2 ml per 10 kg of body weight to the pregnant sows on the 80th day of gestation (DG). On the 105th DG, 50 mg of medroxyprogesterone acetate (Depo-Promone; Pfizer Manufacturing Belgium, Puurs, Belgium) was i.m. administered. Hysterectomies were performed on the 112th DG. The sow was i.m. injected with 2 mg/kg azaperone (Stresnil; Elanco Animal Health, Vienna, Austria) and ketamine hydrochloride 10 mg/kg (Calypsol; Gedeon Richter Plc., Budapest, Hungary) and subcutaneously (s.c.) with 1 mg of atropine sulfate (Atropin Biotika; HBM Pharma, Martin, Slovakia). This treatment was followed by an inhalation of 1–3% isoflurane (Isoflurane; Piramal Healthcare UK Ltd., Morpeth, UK), carried by a mixture of O_2_ and N_2_O. The anesthetized sow was fixed in recumbency on a surgery table, and a 15 cm-long laparotomy incision through the linea alba was made. The uterus, with piglets, was cut with forceps and moved through a 6% chloramine bath into a post-surgery isolator. The uterine wall was cut and the piglets were taken out; the umbilical cords were ligated and cut. The piglets were reversed to a head-down position to remove the remaining amniotic fluid from the lungs. If necessary, breathing and heart action-supporting chest massage was performed. Afterward, the revitalized piglets were transferred to a breeding isolator. The piglets were i.m. treated with 50 mg dextraferranum (Ferribion; Bioveta, Ivanovice na Hane, Czech Rep.) and 5 mg of phytomenadione (vitamin K) (Kanavit; HBM Pharma, Martin, Slovakia), shortly after the hysterectomy.

The piglets were reared in positive-pressure sterile fiberglass isolators with a partially heated floor at 32–35°C. They were fed to satiety 7–8 times a day with an autoclave-sterilized cow’s milk-based formula prepared from condensed milk (Mlekarna Hlinsko, Hlinsko, Czech Republic), using a bottle with a nipple. An initial feeding was done via dropper, placing the milk at the root of the tongue to stimulate swallowing.

### Preterm Germ-Free Piglets

Hysterectomies of pregnant sows were performed between the 102nd and 104th DG in the same manner as the term piglets. If necessary, more frequent feeding and additional nursing care were performed for the preterm piglets to maintain the health of the animals.

### Microbiological Status of the Germ-Free Piglets

Specimens of umbilical cord, amniotic membrane, smears from the body surface, and swabs of mouths of the piglets were collected immediately after hysterectomy. Subsequent microbiological testing was performed twice a week by culturing rectal swabs and smears collected from the piglets in the isolator. All specimens were cultivated to test for the presence of aerobic and anaerobic bacteria, molds, and yeast. The rectal swabs were also stained using Gram’s method.

### Intestinal Lavages

The piglets were euthanized by cardiac puncture under isoflurane anesthesia. 40 cm of the ileum and the distal jejunum were washed with 2 ml of Dulbecco’s phosphate buffered saline (DPBS; Life Technologies, Carlsbad, CA, USA). The whole colon was cut to small pieces in 4 ml of DPBS, gently mixed, and the supernatant taken for a following processing. Aliquots of both washes were taken for bacteria counting, and afterward, the protease inhibitor cocktail (Roche Diagnostics, Manheim, Germany) was added as recommended by the manufacturer. The samples were spun at 1,500 × *g* for 30 min at 8°C, and the supernatants filtered through a 0.2 µm cellulose acetate syringe filter (Sartorius, Goettingen, Germany), then aliquoted and stored at −40°C until analysis using xMAP Technology.

### Blood Plasma

Blood samples containing citrate were centrifuged at 1,200 × *g* for 10 min at 8°C. Protease inhibitor cocktail (Roche Diagnostic) was added to all samples before they were aliquoted, frozen, and then kept at −40°C until used.

### Body Weights and Blood Leukocyte Count

One-week-old piglets were weighed before autopsy. 50 µl of citrated blood in Turk’s solution was used to manually obtain a total leukocyte number in a Bürker’s chamber under an Olympus CX21 light microscope (Olympus, Tokyo, Japan).

### Intestinal Histology

A sample of terminal ileum was fixed in Carnoy’s fluid for 30 min, dehydrated, and embedded in paraffin. 5 µm tissue sections were cut on a Leica microtome RM2245 (Leica Microsystems, Wetzlar, Germany), stained with hematoxylin–eosin, and examined under an Olympus BX 40 microscope with a digital camera Olympus Camedia C-2000 (Olympus). A total of 10 sections from the terminal ileum were analyzed per animal for morphometric parameters (villus length, crypt depth, ratio of villus length to crypt depth, and lamina muscularis thickness). Ten well-oriented villi and crypts, and 10 evenly spaced radial lamina muscularis widths were measured and evaluated in each section using Quick Photo Micro 2.3. software (Promicra, Prague, Czech Republic). A total of five piglets per group from three different hysterectomies (preterm and term piglets, respectively) were analyzed in a blinded fashion.

### Immunofluorescence

Specimens for immunofluorescence observation were embedded in Tissue-Tek (Sakura, Tokyo, Japan), snap frozen in isopentane cooled in liquid nitrogen vapor, and stored at −70°C for further processing. 5-µm acetone-fixed cryosections on SuperFrost/Plus slides (Thermo Fisher Scientific, Darmstadt, Germany) were kept at −40°C until labeling. Sections were incubated with normal rabbit serum (Life Technologies, Carlsbad, CA, USA) for 1 h at RT. Labeling by anti-human claudin-1 and anti-human occludin rabbit polyclonal antibodies (both Life Technologies) was performed overnight at +4°C. The sections were incubated with secondary antibody, Alexa Fluor 488 goat anti-rabbit IgG (Life Technologies), for 2 h at RT. The sections were subsequently embedded in ProLong Gold Antifade Reagent (Life Technologies). Control sections were treated in the same way, with the omission of primary antibody.

### Total RNA Purification and Reverse Transcription

10 mg pieces of intestinal cross sections or mesenteric lymph nodes were stored in RNAlater (Sigma-Aldrich, St. Luis, MO). They were transferred to 2 ml Eppendorf tubes containing 350 µl of RLT Plus buffer of the RNAeasy Micro Plus kit (Qiagen, Hilden, Germany), 2 µl of antifoaming reagent DX (Qiagen), and 2 mm zirconia beads (BioSpec Products, Bartlesville, OK, USA). The samples were homogenized in the TissueLyser LT beadbeater (Qiagen) at 50 Hz for 8 min. The remainder of the total RNA purification followed the RNAeasy Micro Plus kit manufacturer’s instructions. The purity of the RNA was measured in 10 mM Tris-HCl buffer, pH 7.5, as a ratio of absorbances at 260 and 280 nm corrected at 320 nm. 500 ng of total RNA (with A_260_–A_320_/A_280_–A_320_ ≥ 2.00) were used for reverse transcription with the QuantiTect Reverse Transcription kit (Qiagen). The RNA template was preincubated at 42°C for 2 min with a genomic DNA wipeout buffer. The synthesis of cDNA was carried out using a mixture of random n-mers and oligo (dT) primers incubated at 42°C for 20 min. The reaction was stopped by heating the mixture at 95°C for 3 min. 180 µl of PCR-grade water (Life Technologies, Carlsbad, CA, USA) was added to 20 µl of the cDNA mixture to prepare cDNA template for quantitative PCR.

### Real-time PCR

2 µl of cDNA template were added to 18 µl of the FastStart Universal Probe Master (Roche Diagnostic, Manheim, Germany) containing 100 nM LNA probe (Roche Diagnostic) and 500 nM each of the forward and reverse primers to quantify specific sequences in the cDNA templates. The LNA probe-based real-time PCR systems that were used are listed in the Table 1. PCR was performed using the iQ5 real-time PCR cycler (Bio-Rad, Hercules, CA, USA) at 95°C, 10 min (1 × ); followed by 45 cycles of 95°C, 15 s; and 60°C, 60 s. All samples were tested in duplicate. GenEx Pro 6 software (Multid Analyses AB, Gottenberg, Sweden) was used for the normalization of transcripts against β-actin, and cyclophilin A for the counting of their relative expressions.

**Table 1:** Locked nucleic acids based real-time PCR systems used in analyses.

Gene	GenBank acces	Forward primer	#LNA probe
		Reverse primer	
β actin	U07786	TCCCTGGAGAAGAGCTACGA	9
		AAGAGCGCCTCTGGACAC	

Cyclophilin A	NM214353	CCTGAAGCATACGGGTCCT	48
		AAAGACCACATGTTTGCCATC	

Claudin-1	NM001244539	CACCACTTTGCAAGCAACC	3
		TGGCCACAAAGATGGCTATT	

Occludin	U79554	AAAGAGCTCTCTCGACTGGATAAA	42
		AGCAGCAGCCATGTACTCTTC	

TLR2	NM213761	CTGCTCCTGTGACTTCCTGTC	40
		AGGTAGTTCTCCGGCCAGTC	

TLR4	AB188301	CCATGGCCTTTCTCTCCTG	33
		TCAGCTCCATGCATTGGTAA	

TLR9	NM213958	CAATGACATCCATAGCCGAGT	3
		CGTTGCCGCTAAAGTCCA	

MyD 88	AB292176	GCAGCTGGAACAGACCAACT	41
		GTGCCAGGCAGGACATCT	

TRIF	KC969185	ATCTCCCTGGAGGCACTGA	49
		GCTGTCTACACCAGCCCACT	

NLRP3	NM001256770	ACTGCAGCCTCACATCACAC	3
		CGCAGGCTCTGGTTAGAAGT	

RAGE	NM001123218	CCCAACCCACCTTCTCCT	41
		CACAGGCTCCCAGACACTG	

MD2	AB086377	GCTCTGAAGGGAGAGACTGTG	12
		TTGTCCCGGAGAAAATCGTA	

CD14	AB267810	TCTCACCACCCTGGACCTAT	23
		AACTTGCGCGGACAGAGA	

### Protein Extraction and Western Blotting

10 mg of colon (stored at −70°C) were homogenized in 200 µl of Cytobuster protein extraction reagent (Merck, Darmstadt, Germany), with protease and phosphatase inhibitor cocktails added (Roche Diagnostics) in TissueLyser LT (Qiagen). After centrifugation at 18,000 × *g* for 10 min at 4°C, the supernatant protein concentration was determined by a BCA protein assay kit (Thermo Fisher Scientific). 10 µg of total protein in LDS sample buffer (Thermo Fisher Scientific) was resolved on 4–12% Bis–Tris gel (Thermo Fisher Scientific) at 180 V for 30 min and transferred onto a nitrocellulose membrane in an iBlot gel transfer device (Thermo Fisher Scientific). The membranes were blocked in TBST (10 mM Tris-buffered saline, pH 7.2 with 0.1% Tween-20) with 5% skim milk (Beckton Dickinson, San Diego, CA, USA) for 1 h at RT. The TBST-washed membrane was incubated overnight in a mixture of primary rabbit polyclonal anti-claudin-1 antibodies (Thermo Fisher Scientific) diluted 1:500, and anti-β-actin rabbit monoclonal antibody (Cell Signaling Technology, Danvers, MA, USA) diluted 1:10,000 in 5% BSA (Merck) in TBST. The TBST-washed membrane was incubated with a horseradish peroxidase-conjugated anti-rabbit IgG secondary antibody (Jackson ImmunoResearch Europe, Suffolk, UK) diluted 1:10,000 in TBST with 5% skim milk for 1 h at RT. After washing in TBST, the immunoreactive protein bands were developed by the chemiluminescent substrate Clarity (Bio-Rad) and scanned by a C-DiGit blot scanner with Image Studio version 3.1 software (LI-COR, Lincoln, NE, USA). The amount of claudin-1 was normalized with β-actin as a reference protein.

### xMAP Technology

The frozen ileum and colon lavage samples were gradually thawed at 4°C, gently mixed by repeated inversion of the tube, and spun at 10,000 × *g* for 10 min at 5°C. IFN-α, IL-1β, IL-4, IL-6, IL-8, IL-10, IL-12p40, TNF-α, and IFN-γ in intestinal lavages were measured by xMAP technology (Luminex Corporation, Austin, TX, USA) on the Bio-Plex Array System (Bio-Rad) using a Porcine ProcartaPlex kit with magnetic beads (Affymetrix, Santa Clara, CA, USA). The beads were washed on a HydroFlex washer (Tecan, Groedig, Austria) and labeled according to the manufacturer’s instructions. The results were evaluated using Bio-Plex Manager software 4.01 (Bio-Rad).

### ELISA

High mobility group box 1 levels were evaluated in the ileum lavage samples and plasma. HMGB1 quantities were analyzed using the higher sensitivity protocol, according to manufacturer’s instructions (IBL International, Hamburg, Germany). The absorbances were measured at 450 and 620 nm with an Infinite M200 microplate reader, and the results were evaluated with Magellan 6.3 software (Tecan).

### Statistical Analysis

Each set of values was evaluated by the Kolmogorov–Smirnov test for Gaussian distribution. The sets with a normal distribution were evaluated by the unpaired *t*-test to compare mean values using InStat 3 (GraphPad Software, San Diego, CA, USA). The Man–Whitney nonparametric test was used if the distribution was not normal. Differences were labeled as statistically significant at *P* < 0.05 (*), *P* < 0.01 (**), or *P* < 0.001 (***). Results presented in graphs by GraphPad Prism 6 (GraphPad Software) are depicted as a mean + SEM. Body weights, blood leukocyte counts, intestinal morphometry, and HMGB1 levels in the text are expressed as mean ± SD.

## Results

### Preterm and Term Germ-Free Piglets

We performed hysterectomies in pregnant sows on the 112th DG, which is an expected spontaneous delivery term for the miniature pig breed used. An average DG of the preterm piglets was the 103rd DG, which is 92.0% of the expected full term. During the 1-week experiment, 83.9% of the preterm piglets survived. The term piglets were clear of amniotic membrane or bristles, had open eyes, and were running across the isolator immediately after the post-hysterectomy treatment. In contrast, the preterm piglets had tightly attached amniotic membrane on some parts of the body, rare fine bristles, convoluted cloven hoofs, and closed eyes (Figure S1 in Supplementary Material), and their movements were shaky. The milk diet was delivered *via* dropper onto the root of the tongue of the piglets to initiate swallowing. While the term piglets accepted the milk diet by sucking the nipple of a bottle in the 24 h following the hysterectomy, the preterm piglets needed 48 h and longer assistance to learn to accept the diet from the nipple of a bottle. The preterm piglets accepted approximately 1 ml, and the term piglets 1–3 ml per feeding during the first 24 h. The preterm piglets needed increased feeding frequency during the first 3 days to maintain animal health. In the majority of the preterm piglets, the eyes opened on the third day post-hysterectomy, but some of them had closed eyes even on the fifth day post-hysterectomy.

Average body weights of 1-week-old germ-free piglets at the end of the experiments varied in a statistically significant manner (*P* < 0.05) and were 415.5 ± 74.1 g in the preterm and 543.3 ± 146.1 g in the term piglets.

### Blood Leukocyte Counts

Blood leukocyte counts were 3.27 ± 1.12 × 10^9^/l in the preterm piglets and 4.90 ± 0.42 × 10^9^/l in the term piglets. This difference was highly statistically significant (*P* < 0.01).

### Intestinal Histology

**Figure 1:**
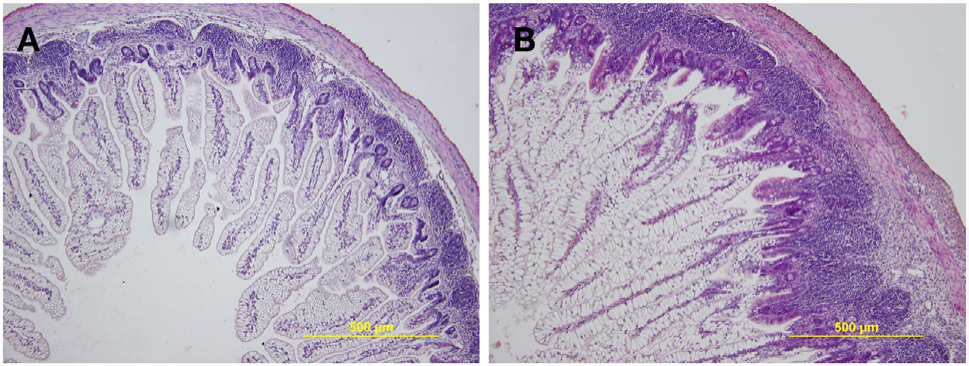
Representative hematoxylin and eosin-stained cross sections of the ileum in the preterm germ-free and term germ-free piglets. The villi in the ileum of the preterm piglets **(A)** are shorter, the Peyer’s Patches are smaller, and the cellularity of the lamina propria mucosa and submucosa is lower in comparison with their term counterparts **(B)**. A total of five piglets per group were analyzed. Magnification: ×100.

The preterm piglet ileum (*n* = 5) displayed statistically significant (*P* < 0.05) shorter villi than term piglets (*n* = 5) (474.2 ± 93.8 vs. 706.0 ± 148.4 µm) and less distinct lymphatic tissue in submucosa (Figure 1). The differences in the crypt depth were not statistically significant (78.3 ± 6.5 vs. 88.1 ± 10.7 µm). The ratio of villus length to crypt depth (5.96 ± 1.01 vs. 8.60 ± 0.60) showed highly statistically significant differences (*P* < 0.01). Stratification of layers was comparable to those of the term piglets, but the layers in the preterm piglets were thinner, especially the muscle layer that statistically significant differed (*P* < 0.05; 40.5 ± 8.5 vs. 56.8 ± 11.4 µm). Enterocytes in both groups were vacuolated, light and transparent. The vacuoles pressed the nuclei that appeared smaller and more basophilic, but the nuclei do not dislocate from the basal pole of the cell in the preterm piglets, as seen in the term piglets. Remarkable differences were visible in the lamina propria mucosa and submucosa that showed decreased cellularity in the preterm piglets, and the tissues appeared to be underdeveloped and similar to fetal tissue, i.e., fibrous tissue is relaxed, sparse, gelatinous, and containing immature light mesenchymal elements.

### Tight Junction Proteins Claudin-1 and Occludin

**Figure 2:**
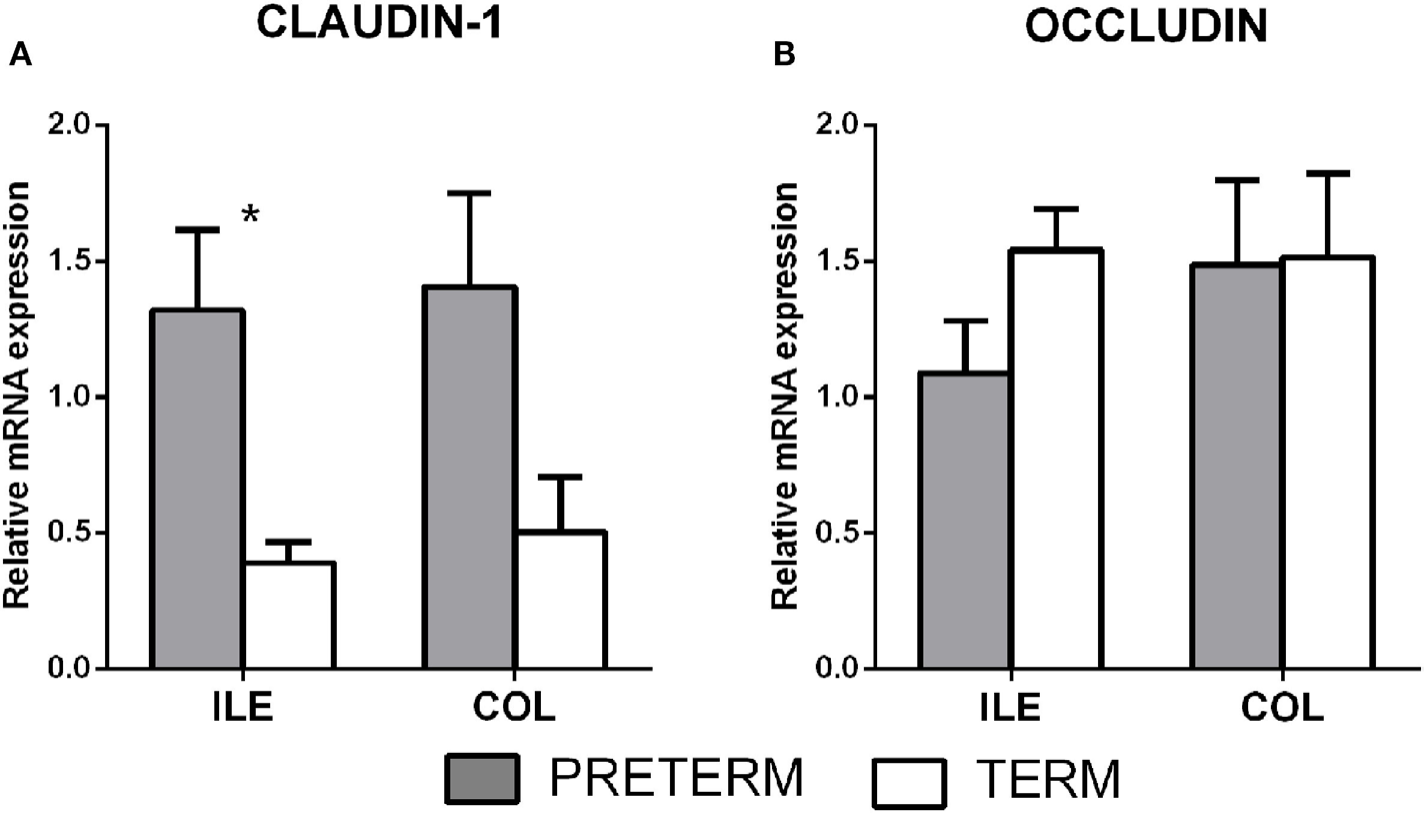
Transcription of claudin-1 and occludin in the intestine of the preterm and term germ-free piglets. The preterm piglets (*n* = 10) are shown in the gray column, and the term piglets (*n* = 6) by the white column **(A,B)**. The results are presented as the mean + SEM. The statistically significant differences in claudin-1 transcription in the ileum are denoted with an asterisk (*P* < 0.05).

Gene transcription of the tight junction proteins claudin-1 and occludin were measured in the terminal ileum and the transversal colon by RT-qPCR. The first (gray) column represents the preterm piglets, and the second (white) column represents the term piglets (Figures 2A,B, respectively). Transcription of claudin-1 was higher in the preterm group in both parts of the intestine (Figure 2A), but the differences were statistically significant (*P* < 0.05) in the ileum only. None of occludin transcription differences was statistically significant (Figure 2B).

**Figure 3:**
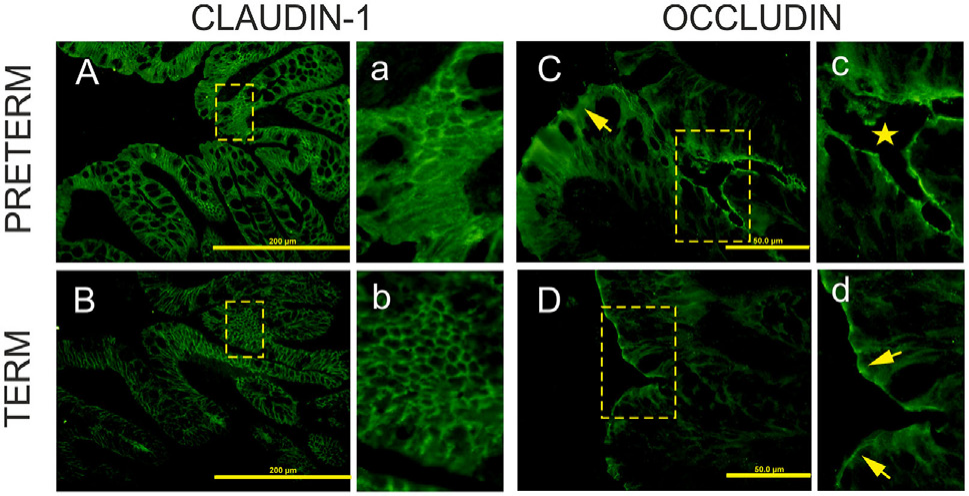
Immunofluorescent localization of claudin-1 and occludin in the colon of the preterm and term germ-free piglets. Cryosections of the colon of the preterm [**(A,C)**a,c] and the term [**(B,D)**b,d] germ-free piglets were stained for the tight junction proteins claudin-1 [**(A,B)**a,b] and occludin [**(C,D)**c,d]. Scale bars for claudin-1 **(A,B)** and occludin **(C,D)** are at 200 µm and 50 µm, respectively. Panels with small letters represent magnifications of ×2 for claudin-1 (a,b) and ×4 for occludin (c,d) of the selected area (outlined with dashed lines) in the corresponding panels denoted with capital letters. Claudin-1 is expressed uniformly from crypt to surface in epithelial lateral membranes in the colon of both piglet groups [**(A,B)**a,b]. In the preterm enterocytes, it is also extensively expressed in the cytoplasm (a). Occludin is expressed at the apical lateral membrane of crypt epithelial cells (c, star), but not in surface epithelial cells in the preterm germ-free piglets [**(C)**, arrow]. It is expressed at the apical lateral membrane of crypt cells (not shown here) and at surface epithelial cells in the term piglets (d, arrows). Sections from five piglets were examined for each group.

Claudin-1 and occludin immunofluorescence-stained patterns were different in each group. Claudin-1 stained continuously from crypt to surface of the epithelial lateral membranes in both groups (Figures 3A,Ba,b). In the colon of the preterm piglets, claudin-1 was also found in the cytoplasm (Figures 3Aa). Occludin expression decreased and was limited only to the epithelial apical lateral membrane of the crypt region in the preterm colon (Figures 3Cc), and it was detected in crypts and the surface region in the term colon (Figure 3Dd).

**Figure 4:**
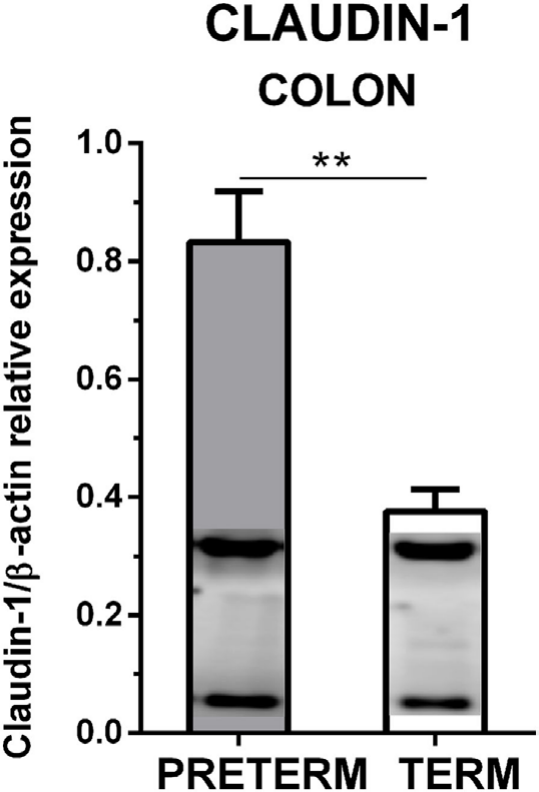
Immunoblotting analysis of claudin-1 in the colon of the preterm and the term germ-free piglets. The expression of claudin-1 (normalized to β-actin) in the colon of the preterm (*n* = 10) and the term (*n* = 6) germ-free piglets was analyzed by Western blot. Examples of β-actin (upper) and claudin-1 (lower) bands detected in one sample from each group are shown in the columns. The results are depicted as mean + SEM. The statistical significances are denoted with double asterisks (*P* < 0.01).

Claudin-1 and β-actin were simultaneously detected by Western blot in the colon tissue. The immunoblots did not show any non-specific bands (Figure 4). Claudin-1 values in the preterm piglets (β-actin normalized) were statistically significantly higher (*P* < 0.01) than in the term piglets.

### Transcription of TLR2, TLR4, TLR9, MyD88, TRIF, Inflammasome NLRP3, RAGE, MD2, and CD14

**Figure 5:**
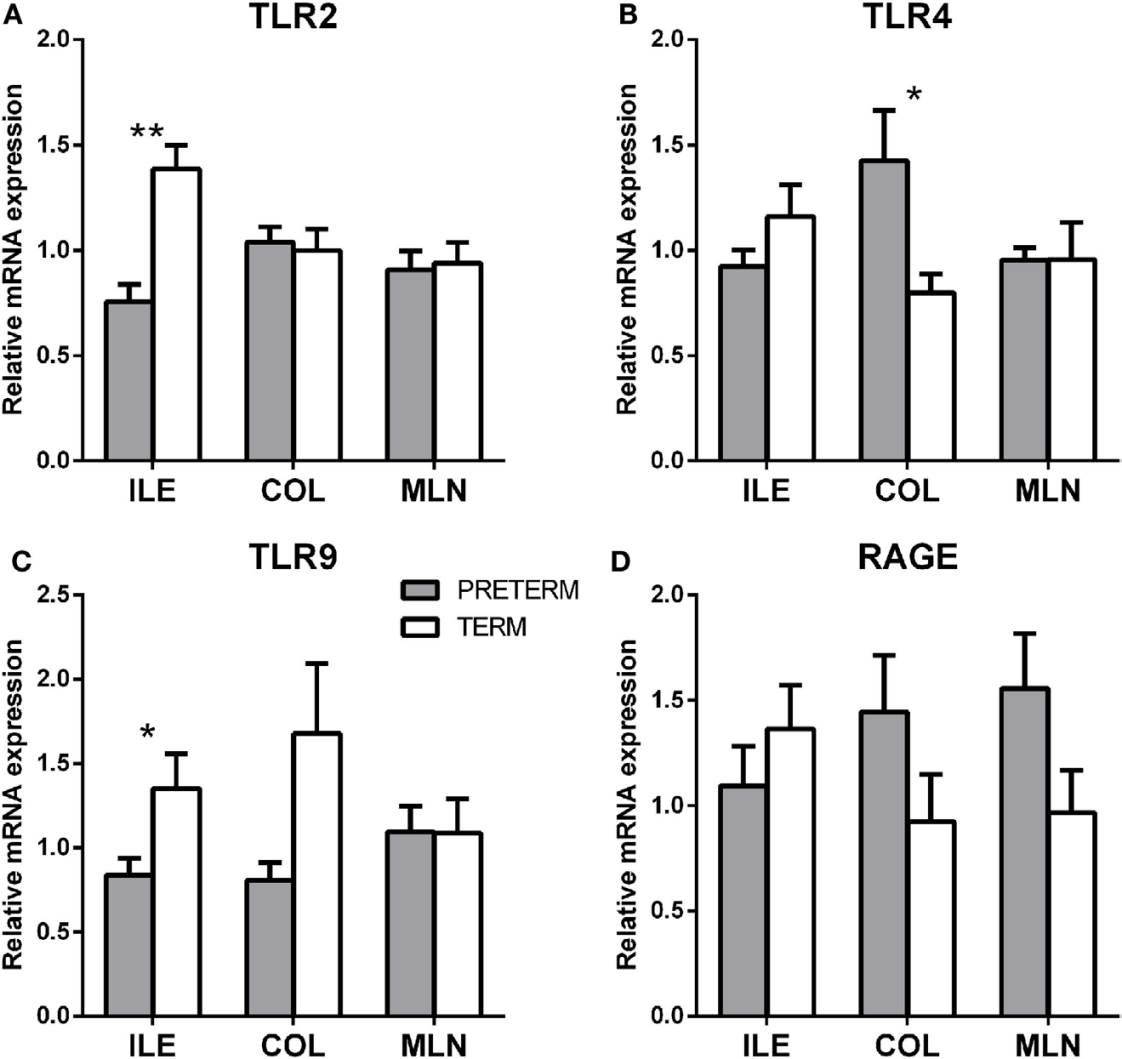
Transcription levels of pattern recognizing receptors TLR2, 4, 9, and RAGE in the intestine and mesenteric lymph nodes of the preterm and term germ-free piglets. Transcriptions of TLR2 **(A)**, TLR4 **(B)**, TLR9 **(C)**, and RAGE **(D)** were compared in the preterm (*n* = 6, gray columns) and the term (*n* = 6, white columns) germ-free piglets in the ileum, the colon, and the mesenteric lymph nodes. The results are presented as mean + SEM. The instances of statistical significance in the colon are denoted with asterisks (*P < 0.05 and **P < 0.01).

Gene transcription of TLR2, TLR4, TLR9, MyD88, TRIF, inflammasome NLRP3, RAGE, and TLR4 coreceptors MD2 and CD14 were measured in the terminal ileum, transverse colon, and mesenteric lymph nodes by RT-qPCR. The first gray column represents the preterm piglets, and the second white column represents the term piglets (Figures 5A–D).

TLR2 (Figure 5A; *P* < 0.01) and TLR9 (Figure 5C; *P* < 0.05) were significantly higher in the ileum of the term piglets, but no significant differences were found in the colon and the mesenteric lymph nodes (MLN). In contrast, TLR4 values were significantly higher (*P* < 0.05) in the preterm group in the ileum (Figure 5B). RAGE (Figure 5D) showed seemingly high differences between groups, but they were not statistically significant.

Other transcripts (MyD88, TRIF, inflammasome NLRP3, MD2, and CD14) showed similar values in the majority of the cases, in both groups, and their differences were without any statistical significance. They were not shown on the Figure 5.

### Interferons, a Chemokine, and Inflammatory Cytokines in the Intestinal Lavages

**Figure 6:**
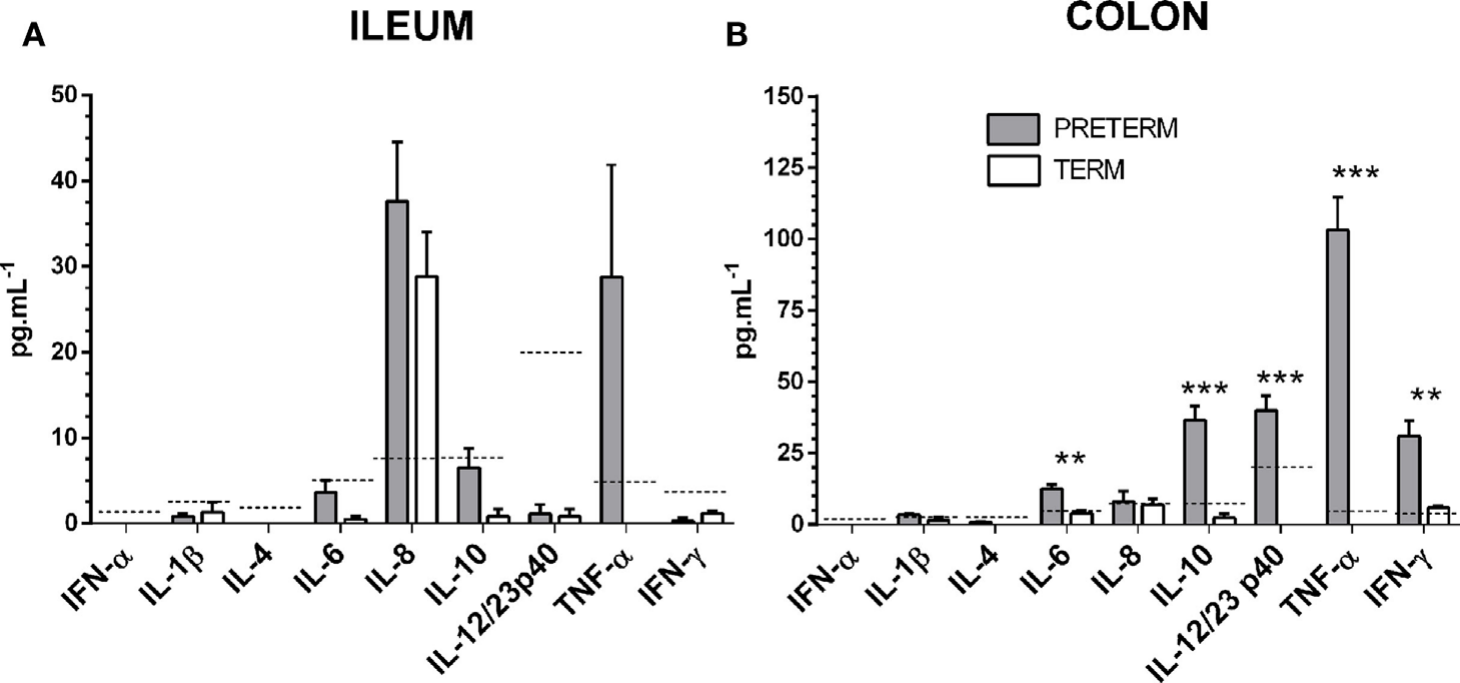
Inflammatory cytokines in the ileum and the colon. Levels of interferon/chemokine/cytokines in the ileum **(A)** and the colon **(B)** of the preterm (*n* = 10, gray columns) and the term (*n* = 6, white columns) piglets were compared. The values in the both the ileum and the colon were comparable or higher in the preterm piglets, but the statistically significant differences were found in the colon only. The results are presented as mean + SEM. The statistical significances in the colon are denoted with asterisks (**P < 0.01 and ***P < 0.001). Dashed lines indicate individual cytokine detection limits.

Levels of interferons α and γ, chemokine IL-8, and inflammatory cytokines IL-1β, IL-4, IL-6, IL10, IL-12/23 p40, and TNF-α were measured in the ileum (Figure 6A) and the colon (Figure 6B) by multiplex assay. Values for several of the proteins were under the detection limit, or very low. IL-6 and IFN-γ were statistically significantly increased (both *P* < 0.01) in the preterm group in the colon. At the same time, IL-10, IL-12/23 p40, and TNF-α were increased in a statistically significant manner (all *P* < 0.001) in the preterm group in the colon. Relatively high levels of IL-8 were found in both groups of piglets, in the ileum and lower in the colon but without statistical significance between the preterm and term groups. Levels of TNF-α showed a relative increase in both parts of the intestine in the preterm group, but the increase was only statistically significant in the colon.

### Local and Systemic HMGB1 Levels

Levels of HMGB1 in both the ileum and plasma were higher in the preterm piglets (Figure S2 in Supplementary Material). Differences in local or systemic values, however, were not statistically significant.

## Discussion

Colostrum-deprived piglets could serve as a model of preterm immunocompromised infants, given the deprivation of the mother’s immunoglobulins, cytokines and growth factors, and cells (15, 35, 36). The deprivation of the postnatal modulation of the GIT by biologically active compounds from colostrum and 2-days parenteral nutrition are considered potential triggers of NEC in the piglet model of NEC (14, 37). NEC is a multifactorial disease that affects approximately 7% of preterm infants weighing <1,500 g (38). No obvious signs of NEC occurred in our experiments. It underscores the possible impact of microbiota in the pathogenesis of this illness.

The enterocytes in the intestine of piglets in the early postnatal period contain an apical canalicular system, creating large vacuoles in the ileum that enable transfer of colostral immunoglobulins from the intestinal lumen across the intestinal epithelium, with retention of the biological activity. These vacuoles disappear in conventional piglets around 2 weeks of age (39). Changes in enterocytes are related to absorption of macromolecules, especially immunoglobulin G, from colostrum (17). The influence of microbiota on the accelerated maturation of enterocytes was shown in experiments with gnotobiotic piglets (19), and the specific microbiota-driven maturation of the enterocytes in monocolonized gnotobiotic piglets was also reported (40). In our experiments, the enterocytes in both groups of the germ-free piglets showed large vacuoles in 1-week-old germ-free piglets (Figure 1). Additionally, the ileum of the preterm germ-free piglets showed further markers of immaturity, including a lack of displacement of the enterocyte nuclei, less distinct lymphatic tissue in the submucosa, and relaxed fibrous tissue containing light mesenchymal elements in comparison to their term counterparts. These differences show the similarity of the ileum in preterm piglets to that of the fetal developmental period.

The enterocytes, with desmosomes, adherent, gap, and tight junctions (TJs), create a semipermeable barrier. Disruption of this barrier leads to “leaky gut” syndrome (41). The permeability of the intestinal barrier has been studied using different principles and can allow for the detection of different molecules, e.g., perorally applied lactulose-mannitol detected *via* the urinary lactulose-to-mannitol ratio (42) or an FITC-dextran leak from the intestine to plasma (43). These tests are relatively simple and reflect the functionality of the intestinal barrier. Both methods, however, suppose normal peristalsis. Therefore, potential aperistalsis in preterm newborns or enteric infections limit their use, and other methods would have to be used. One of them is the plasma detection of an intestinal fatty acid binding protein (iFABP) because it is produced directly in the small intestine and its intestinal presence is independent on the peristalsis (14).

Tight junctions join adjacent enterocytes in their apical part, and this barrier separates the host body from the intestinal lumen that harbors a dense microbial community (4). TJ proteins contain several groups of distinct proteins, e.g., claudins, occludin, zonula occludens, and others that selectively permit the absorption of nutrients and water *via* paracellular pathways, and protect the host against penetration of bacterial toxins and bacterial translocation from the intestine to the body (44). In mammals, claudins comprise a group of at least 27 proteins with different properties. The majority of them prevent paracellular transport (e.g., claudin-1 and 4), though a few of them facilitate it (e.g., claudin-2 and 10) (45). Increased expression of claudin-1, but not occludin, in jejunal mucosa was observed in the piglet groups in which diarrhea (caused by enterotoxigenic *E. coli*) was reduced by colistin or plant extract (46). We evaluated the intestinal barrier protein changes of the preterm and term piglets by the transcription and expression of two representative TJ proteins, claudin-1, and occludin. Tight junctions are dynamically regulated structures that are modulated by various stimuli, including inflammatory cytokines. IL-6, IL-10, IL-12/23 p40, TNF-α, and IFN-γ in the colon were increased in our preterm germ-free piglets (Figure 6). The fetus is not endangered by the loss of electrolytes in the prenatal period because it develops in the amniotic fluid and swallows it, thereby replenishing its electrolyte supply (2). We hypothesize that the higher transcription of claudin-1 in the preterm piglets in our experiments demonstrated the adaptation of the intestinal barrier of the underdeveloped intestine to protect the body against the loss of electrolytes in the sudden change of conditions after delivery. The lower transcription level of claudin-1 in the term group ileum (Figure 2A) could reflect an ontogenetic adaptation of the intestine of the term piglets for postnatal life. An intracellular distribution of claudin-1 in the colon (Figures 3Aa) and its higher levels in the colon tissue (Figure 4) document its substantial expression in the preterm piglets. In contrast to the increased transcription of claudin-1 in the preterm group, the transcription of occludin was in both groups was comparable (Figure 2B). While claudin-1 is related to transport of electrolytes, occludin plays a role in sealing the intestinal barrier against the transfer of macromolecules and cells (47). Additionally, the difference in fluorescent-staining distribution for claudin-1 and occludin protein in the colon of the term and the preterm piglets suggests the absence of fully functional TJ complexes in the preterm piglets (Figure 3).

Both groups, the preterm and the term germ-free piglets, showed the low peripheral blood leukocyte counts typical for germ-free piglets. These counts reflect an absence of microbial stimulation in sterile conditions, and they would be much higher in microbe-stimulated conventional piglets (48). Theories dealing with the permeability of the intestinal barrier, bacterial translocation, sepsis, the role of mesenteric lymph nodes in these processes, and the relation to Matzinger’s danger theory (49) and sterile inflammation (50) have been developed (51). This is also why we included mesenteric lymph nodes in our studies. The absence of microbial stimuli in the germ-free piglets quite logically turns our attention to sterile inflammation. The DAMPs, often synonymously termed alarmins, are molecules specific to the host organism that are normally hidden to immune recognition. The release of the DAMPs and their availability to the host’s immune recognition signals cell damage, similar to the presence of PAMPs. There is an overlap of PRRs, including TLRs, that recognize both PAMPs and DAMPs (28). Therefore, systemic inflammatory response syndrome (SIRS) in the case of sterile inflammation leads to similar consequences as sepsis (30). An important representative of the DAMPs is a nuclear DNA-binding HMGB1 protein. While cell necrosis leads to the release of HMGB1 and an inflammatory reaction, apoptosis as a mechanism of physiological tissue maturation results in the release of HMGB1 that is covered in apoptotic bodies and does not induce the inflammation (52). It can be also produced by immune cells after by their stimulation (31). HMGB1 is abundantly secreted and/or released in the intestinal tissues of human inflammatory bowel diseases patients, and fecal HMGB1 was recognized as the marker for intestinal inflammation (53). Therefore, fecal HMGB1 has recently been suggested as a novel noninvasive biomarker of intestinal inflammation in pediatric and adult patients (54). Gnotobiotic piglets that suffered from enteric infection showed increased levels of HMGB1 in the intestine. Its levels were related to the severity of the infection (20). The similarity of the consequences of infections and tissue damage can explain the fact that HMGB1 (and other alarmins, at a lower level) is a ligand for receptor advanced glycation endproducts (RAGE), but also for TLR2, TLR4, and TLR9 (50). HMGB1 is a late mediator of inflammation and amplifies inflammatory reactions that may lead to organ dysfunction (55). A relationship between increased transcription of TLR4, intestinal maturation, and NEC was described (56), as well as the association of NEC with increased intestinal expression and serum HMGB1 levels, and HMGB1-driven inhibition of enterocyte migration in a TLR4-dependent manner (57). Based on this knowledge, we expected that in our experiments TLR-mediated signaling in sterile conditions should reflect a possible HMGB1 release that blocks enterocyte maturation and/or turnover. To fully analyze our results with regard to this expectation, we examined both TLR4 alternative signaling pathways *via* adaptor molecules MyD88 or TRIF, and TLR4 coreceptors MD2 and CD14. The local ileal and systemic HMGB1 values were indeed higher (but statistically non-significantly) in the preterm group (Figure S2 in Supplementary Material), but all levels of transcription for HMGB1 receptors (RAGE, TLR2, TLR4, and TLR9) were unexpectedly lower in these piglets, and we were unable to elucidate the reason for the exception in the case for TLR4 levels in the colon. We suppose that this incongruity with our expected outcome would be clarified by the detection of soluble decoy receptors that can disrupt receptor triggering to prevent overactivation or dysregulation of the innate immunity (58, 59).

Inflammasomes create a group of cytosolic protein complexes that mediate host immune response to microbial attacks and tissue damage. Members of the NLR family NLRP1, NLRP3, and NLRC4, and the cytosolic receptor AIM2 link microbial and endogenous danger signals to the activation of pro-caspase-1 to caspase-1 that cleaves pro-IL-1β and pro-IL-18 to mature, biologically active IL-1β and IL-18, respectively (60). The NLRP3 inflammasome is sensitive to PAMPs and alarmin HMGB1, uric acid crystals, ATP, β-amyloids and cholesterol crystals (50). NLRP3 transcription in the ileum, colon, and mesenteric lymph nodes were nonsignificantly statistically higher in the term piglets in all cases. The nearly equivalent values in both piglet groups negate any speculations on the role of inflammasome NLRP3 in the early postnatal development of the preterm and the term gnotobiotic piglets. Inflammasomes, mainly the NLRP3 inflammasome, play a role in inflammatory processes, many diseases and metabolic states, including Multiple Sclerosis, Alzheimer’s disease, Parkinson’s disease, atherosclerosis, type 2 diabetes, and obesity (61).

Cytokines regulate many biological processes, including pregnancy, inflammation, and sepsis (55, 62). While low and intermediate cytokine levels fulfill their physiological functions, high levels manifest as a “cytokine storm,” resulting in an excessive innate immune response that is harmful to the host (59). We speculate that in the case of the preterm group, intestinal underdevelopment may increase the release of inflammatory cytokines, but in levels appropriate for the physiological role of cytokines. Minute or non-detectable levels of IL-1β in both piglet groups (Figure 6) correlated with no increased activation of transcription of the NLRP3 inflammasome between the groups, suggesting that the maturity of the intestine in the preterm piglets did not influence the activity. Increased levels of IL-6 may indicate mild inflammation in the intestine of the preterm piglets and are consistent with increased levels of immunoregulatory IL-10, and pro-inflammatory TNF-α in the intestine. In the colon of the preterm piglets, increased levels of p40 IL-12 and the IL-23 shared unit, and IFN-γ were also observed. IL-6 governs the production of acute phase proteins, and together with IL-1β, IL-12/23/p40, and TNF-α, are termed proinflammatory cytokines and are induced in intestinal mucosa during sepsis, and in enterocytes after *in vitro* treatment with endotoxin and other proinflammatory cytokines (63). Noteworthy were the relatively high levels of IL-8 in the ileum of both piglet groups. IL-8 was found sustainedly transcribed and expressed along the human fetal intestine during its gestational maturation(64). IL-8 is a chemokine with its main function to attract neutrophils into inflammatory sites and activate them (65). Increased serum IL-8 levels were described in NEC infants, and high IL-8 levels were proposed as a diagnostic marker for NEC (66). Neither in the ileum nor in the colon was neutrophil infiltrations found in any groups. IL-8 levels were increased in the distal small intestine tissue of the preterm piglets shortly after the introduction of an infant formula diet (TGF-β) and were higher in the small intestine compared to the colon (67). Similarly, in our experiments, IL-8 was higher in the ileum than in the colon. Its levels can be induced by the used cow milk-based formula (68), or it naturally occurs in the developing intestine (64).

Cytokines as diagnostic markers are usually detected as systemic in serum/plasma, but their local production more precisely reflects the state of developed or inflamed mucosal surfaces. Unfortunately, such data in humans are scarcely available. Increased levels of serum IL-10 are considered a predictor of severity and fatal outcome (69). Anti-inflammatory IL-10 dampens inflammatory reactions. We found its increased levels in the colon of the preterm piglets. A similar situation is noted in the case of p40 IL-12 and the IL-23 shared unit. All these cytokine levels, with the exception of TNF-α, are potentially considered at “regulatory levels” or levels that are common for a mild inflammatory reaction present on the mucosal surface of the GIT of conventionally balanced microbiota-colonized hosts. Cytokine levels indicating the highly inflamed intestine in the gnotobiotic piglets would be more than one order higher as we saw in infectious experiments with *Salmonella* Typhimurium and *E. coli* O55 infected piglets (20). The presence of the mild inflammatory process in the intestine of the preterm piglets probably predicts increased local and systemic HMGB1 levels. HMGB1 is a late inflammatory mediator that induces the production of other inflammatory cytokines and amplifies the inflammatory reaction (31, 55). Little is known about the impact of HMGB1 on developed intestines. Its relation to intestinal pathology in a rat model of NEC and intestinal specimens of infants suffered from NEC (70), as well as its findings in feces of pediatric patients with IBD (54) suggest increased attention should be paid to HMGB1 and other alarmins, as they relate to the pathophysiology of the developed intestine.

## Conclusion

A full-term prenatal period is necessary for the complete development of human organ systems and the transfer of biologically active compounds from the mother to the fetus. After birth, the GIT is settled by a divergent microbiota that stimulates the immune system. In addition, feeding *via* breast milk containing biologically active factors influences postnatal maturation of the GIT and the health of the infant. If the gestation period is shortened, infants are born with underdeveloped organ systems and lower levels of biologically active molecules, including immunoglobulins.

In contrast to newborn infants, piglets are born without protective maternal immunoglobulins because the epitheliochorial placenta of the pig prevents their prenatal transfer (1, 16). The newborn piglets receive immunoglobulins and leukocytes after birth from colostrum (35). By preventing the intake of colostrum, the newborn piglets become immunocompromised. Such immunocompromised piglets can survive in the microbiologically controlled conditions of gnotobiotic isolators.

We derived preterm germ-free piglets by hysterectomy, and they were subsequently deprived of colostrum intake. The surgery was performed at a duration of gestation that corresponds to 30–32 weeks of human gestation (71). Selected immunological and epithelial barrier characteristics were compared in the preterm and the term germ-free piglets. We concluded that the intestine of the preterm germ-free piglets showed signs of underdevelopment and mild inflammation, as compared to their term counterparts.

To our knowledge, this is the first study using preterm germ-free piglets. These piglets, if colonized with preterm infant microbiota, could provide an animal model of preterm infants. This animal model could be used to study the pathological conditions associated with preterm status, and the effects of various treatments to improve the physiological development of preterm infants.

## Ethics Statement

Experiments with animals were approved by the Animal Care and Use Committee of the Institute of Microbiology in accordance with local and European laws for the protection of animals. The number of piglets in the preterm group was higher to accommodate for unexpected events, but the number of the term piglets was reduced to a number that was sufficient for statistical comparison between groups.

## Author Contributions

AS formulated an original hypothesis, designed the study, performed the experiments, histology, and immunochemistry techniques, analyzed and interpreted results, co-wrote the manuscript, and contributed funding. VS performed the experiments and RT-qPCR analyses. ZS analyzed and interpreted histology. IS designed the study, performed the experiments, analyzed and interpreted RT-qPCR and xMAP technology results, performed statistics, and co-wrote the manuscript. All authors contributed to discussions and reviewed the manuscript before its submission.

## Conflict of Interest Statement

The authors declare that the research was conducted in the absence of any commercial or financial relationships that could be construed as a potential conflict of interest.
