# Supplementary material for: Preterm Life in Sterile Conditions: A Study on Preterm, Germ-Free Piglets
Source: Front Immunol. 2018 Feb 14;9:220. doi: 10.3389/fimmu.2018.00220 (PMC5817058; doi:10.3389/fimmu.2018.00220)
Supplement: Supplementary file 1 [file image_1.PDF]

Supplementary Material

Preterm Life in Sterile Conditions: A Study on Preterm, Germ-Free Piglets

Alla Splichalova, Vera Slavikova, Zdislava Splichalova, Igor Splichal*

*** Correspondence:** splichal@gnotobio.cz

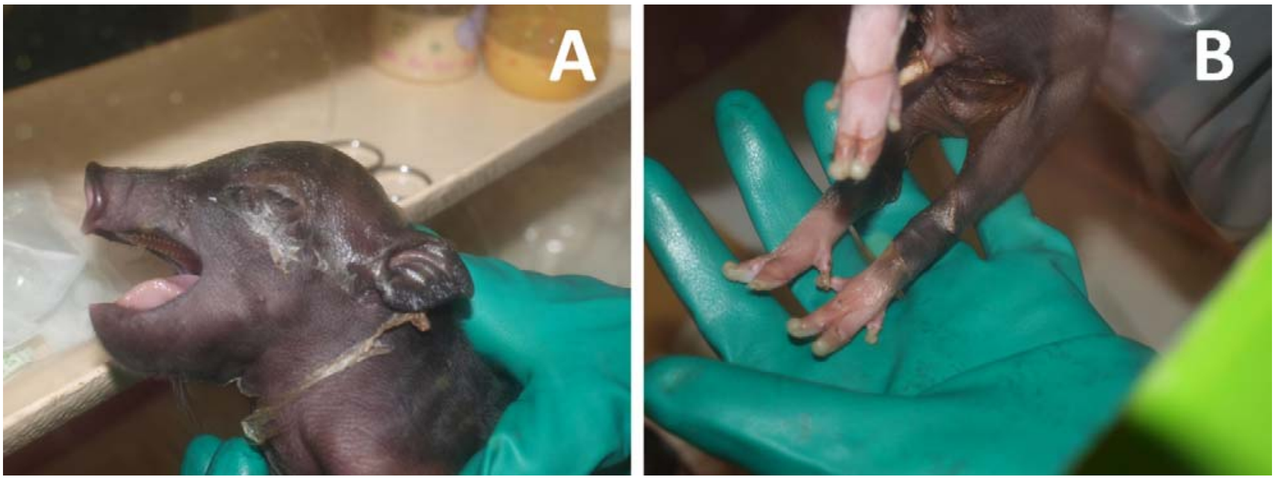

Figure S1. The preterm piglets had closed eyes, tightly attached amniotic membrane on some parts of the body (A), and convoluted cloven hoofs (B).

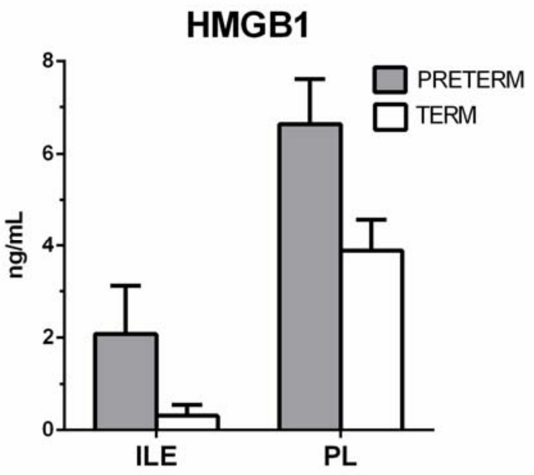

Figure S2. HMGB1 levels in the ileum (ILE) and blood plasma (PL).
Systemic and local levels of HMGB1 were higher in the preterm piglets, but differences between groups were not statistically significant ($n_{PRETERM}$ = 10, n_{TERM} = 6).
